# Information provision and attentive listening as determinants of patient perceptions of shared decision-making around chronic illnesses

**DOI:** 10.1186/s40064-016-3086-4

**Published:** 2016-08-22

**Authors:** Ana-Belén del Río-Lanza, Leticia Suárez-Álvarez, Ana Suárez-Vázquez, Rodolfo Vázquez-Casielles

**Affiliations:** 1 Biomedicine and Health Cluster, Business Administration Department, School of Economy and Business, University of Oviedo, Avenida del Cristo s/n, 33071 Oviedo, Asturias Spain; 2 Spanish Federation of Haemophilia, Madrid, Spain

**Keywords:** Decision making, Patients perspective, Self efficacy, Proactive behaviour, Chronic disease, Patient-centered care, Physician–patient relations, Communication programs

## Abstract

**Background:**

While chronic illnesses are a major concern of the health system worldwide, little is known about patients–physicians communication. Growing demand for patient-centered care and shared decision-making have increased the interest for patients–physicians communication. Based on previous literature, we propose a model in which the effect of information provision and attentive listening over patients’ perceptions of shared decision-making (PPSDM) is mediated by the variables self-efficacy and proactivity. Primary data were collected between April and August 2014 through an online survey of patients with haemophilia. Haemophilia is a chronic disease in which many options of treatment are available. The right option depends, to some extent, on patient’s preferences. In this context, great uncertainty exists when choosing treatment option and shared decision-making plays an essential role.

**Results:**

A total of 181 patients with haemophilia participated in the survey. The psychometric properties of the measurement scales were evaluated by means of a confirmatory factor analysis. A structural equation model was designed. Results show that provision of information and attentive listening determine PPSDM through patients’ self-efficacy and proactivity in requesting information.

**Conclusions:**

It is important to incorporate communication training in medical education, particularly provision of information and attentive listening. These skills help the healthcare professional to gain a deeper understanding of the patient. Furthermore, provision of information and attentive listening are fundamental in helping patients not to undervalue their personal knowledge and expertise in relation to their doctors. These strategies encourage them to adopt a more active position in requesting information. Encouraging a proactive behaviour of patients and their relatives helps them to realize the need to participate and to make them feel that they are part of the decision-making process.

## Background

The World Health Organization (WHO) estimates that by 2020, 60 % of all diseases around the world will be chronic illnesses, and they will cause three-quarters of the world’s deaths. Chronic illnesses are becoming increasingly prevalent (WHO 2011) and leading to rising costs in the economies of the world (McAdam 2013). In this context, a patient-centered health care system seems to be crucial to satisfy the needs of patients with chronic conditions. The goal of this system is threefold (Cramm and Nieboer 2014): (1) to inform the patients to improve the knowledge about their own illness; (2) to activate the patients to increase the roles they assume in the illness management; (3) to promote the interaction between patients and healthcare professionals. Shared decision-making (SDM) appears as an approach able to cope with this necessity of enhancing the quality of care of chronically ill patients and improving the relationships between patients and healthcare professionals (Branda et al. 2013; Siegel et al. 2015).

SDM describes an interaction process in which both patient and doctor participate actively in finding an agreement based on shared information. The objective is to reach a decision in which both patient and physician are involved (Hölzel et al. 2013). Patient participation in health care is seen as an important ethical, legal and social aspiration (Lam et al. 2014). Thus SDM is now widely regarded as an essential component to reduce the asymmetry in information exchange and power distribution between doctor and patient (Charles et al. 1997) and as a means of providing good quality healthcare (Entwistle and Watt 2006) and improving patient satisfaction and treatment adherence (Joosten et al. 2008).

In general, the literature on SDM suggests that patients with chronic illnesses want to be told about treatment alternatives and to be involved in treatment decisions to encourage them to take a more active role in medical decision-making (Hamann et al. 2007; van den Brink-Muinen et al. 2011). But SDM is difficult to apply in practice (Elwyn et al. 2012; Blair and Légaré 2015) and some patients take on a passive role in the decision-making (Tariman et al. 2010; Petriwskyj et al. 2014). This is due to multiple barriers to do with the patient, the healthcare professionals and the institutional framework (Légaré et al. 2006; Frosch and Elwyn 2014; Joseph-Williams et al. 2014). Thus recent work calls for research into the antecedents of SDM (Joseph-Williams et al. 2014; Shay and Lafata 2015). According to Kriston et al. (2010, p. 94) “in contrast to its outcomes, the process of SDM is rather under researched and has not received much attention in the past”.

It is important to analyze patients’ perceptions of SDM (henceforth, PPSDM). Previous research shows that the patient’s perspective of SDM differs from the doctor’s perceptions (Janz et al. 2004; Fiks et al. 2011) and from observer ratings (Burton et al. 2010; Wunderlich et al. 2010). Moreover, in a recent systematic review of SDM, Shay and Lafata (2015) find that when studying the relation between SDM and patient outcomes (such as improved satisfaction and less decisional conflict), the measurement perspective that most often shows a significant, positive relation with patient outcomes is patient self-reports.

With this in mind, our objective with this work is to model the impact of provision of information and attentive listening on PPSDM. We test five hypotheses via structural equation modelling (SEM). This statistical technique is a modelling approach that can be used to simultaneously model the pathways of influence of multiple variables on outcomes of interest.

### Research model and hypotheses

The complexity of patients–physicians communication have led to analyze it from different points of view. At least three dimensions can be considered when approaching it (Tates and Meeuwesen 2001).

#### Relational dimension

It allows to distinguish between instrumental or task focused-behaviour, oriented to the cure, and affective or socio-emotional behaviour, oriented to the care. While asking questions and providing information are essential in a task focused-behaviour, empathy and the ability to show concern are relevant skills in socio-emotional behaviour.

#### Structural dimension

It describes unequal positions between the doctor and the patient in terms of power, autonomy and responsibility in decision making.

#### Linguistic dimension

A dimension related with the content of the interaction. It alludes to the linguistic dimension in medical encounters. It reflects how, due to differences in previous knowledge, different patients can assign different meanings to the same terms.

This paper focuses on the first dimension mentioned, the relational one. Taking into account previous literature, four variables and its effect over PPSDM have been considered: health care professionals’ information provision (PI); health care professionals’ attentive listening (AL); patient’ self-efficacy (SE) and patient’ proactive behaviour (PB).

Following Liang et al. (2002) and Maly et al. (2004) we assume that providing practical advice and correct information that is personalized according to the patient’s individual circumstances is associated with a greater perception of choice and more involvement in the decision-making. What is less researched is how healthcare professionals’ provision of information can influence PPSDM. Oftedal et al. (2010) argue for the need to give higher priority to practical advice and information as a means of stimulating the patient’s self-efficacy—i.e. their expectations of being able to cope with their illness by increasing knowledge, reducing feelings of uncertainty, and enhancing emotional and social adjustment (Carpenter et al. 2010). The first hypothesis of this work is as follows:

##### **Hypothesis 1 (H1):**

Healthcare professionals’ provision of information has a positive effect on patient self-efficacy.

Hartman et al. (2013) show that it is important to listen to the patient and to listen actively, for example summarizing the patient’s beliefs and concerns. Attentive listening is seen as a comprehensive way of dealing with patient orientation because it makes people feel respected, important, intelligent, confident, liked and valuable and creates a trusting atmosphere. For all this, attentive listening environments have been assumed to promote a free and open exchange of ideas and information (King 1978) and improve the doctor–patient relationship (Jagosh et al. 2011). Likewise, Hausman (2004) finds that when the doctor has a personal and close relationship with the patient, and listens to their requirements, a two-way communication ensues from doctor to patient and from patient to doctor. As a consequence, we propose that:

##### **Hypothesis 2 (H2):**

Healthcare professionals’ attentive listening has a positive effect on patient proactivity in requesting information.

The increasing importance of the relational dimension of patients–physicians communication reflects a change from physician-centered care towards patient-centered care. A different concept of patients characterized by higher levels of self-organization and proactivity, underlies this movement. This model implies a change from an approach based on doctors’ power and autonomy to an approach based on patients’ power and autonomy. In this context, patients have to feel a relationship between actions and results (Bandura 1998). Self-efficacy seems to be a crucial factor to increase patients’ proactivity by encouraging them to pose questions, to discuss their preferences and to disagree with recommendations (Kahana and Kahana 2003; Mallin et al. 2014). So the claim could be made that:

##### **Hypothesis 3 (H3):**

Patient self-efficacy has a positive effect on patient proactivity in requesting information.

In parallel, Heckman et al. (2011) argue that self-efficacy is associated with increased participation in treatment decision-making and improved health-related quality of life. Hypothesis 4 tests this relation in the context of this work:

##### **Hypothesis 4 (H4):**

Patient self-efficacy has a positive effect on PPSDM.

Finally, assuming that an individual’s proactive behaviour refers to anticipatory, change-oriented and self-initiated behaviour in different situations, Kahana and Kahana (2003) argue that patients’ proactivity in communicating with their doctors is likely to receive encouragement for expressing their screening preferences. Additionally, patient proactivity in communication involves assertiveness in acting as healthcare consumers and in seeking to take an active role in medical decision-making (Kahana and Kahana 2001). The final hypothesis of this work follows:

##### **Hypothesis 5 (H5):**

Patient proactivity in requesting information has a positive effect on PPSDM.

**Fig. 1:**
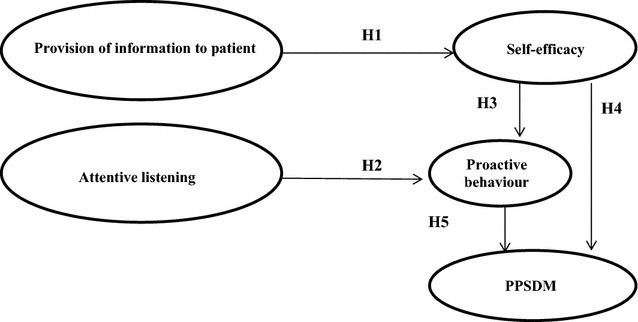
Research model and hypotheses

Based on these previous comments (see Table 1) we propose the model shown in Fig. 1.

**Table 1:** Hypotheses and theoretical basis

Hypotheses	References
H1: Healthcare professionals’ provision of information has a positive effect on patient self-efficacy	Liang et al. (2002)
	Maly et al. (2004)
	Carpenter et al. (2010)
	Oftedal et al. (2010)
H2: Healthcare professionals’ attentive listening has a positive effect on patient proactivity in requesting information	King (1978)
	Hartman et al. (2013)
	Jagosh et al. (2011)
	Hausman (2004)
H3: Patient self-efficacy has a positive effect on patient proactivity in requesting information	Kahana and Kahana (2003)
	Mallin et al. (2014)
H4: Patient self-efficacy has a positive effect on PPSDM	Heckman et al. (2011)
H5: Patient proactivity in requesting information has a positive effect on PPSDM	Kahana and Kahana (2001, 2003)

## Methods

### Study population

Haemophilia is a chronic, congenital illness characterized by a deficiency in a clotting factor—a protein in the blood that controls bleeding. As a result, people with haemophilia can bleed for longer than normal, and some may experience bleeding into joints, muscles, or other parts of their bodies that can lead to joint damage and disability. This disorder, especially in its severe forms, has been associated with mortality and morbidity (Salem and Eshghi 2013). Prophylactic therapy, which involves regular factor replacement (at least three times per week or daily), is of critical importance (Manco-Johnson et al. 2007). Haemophilia is a chronic disease characterized by the existence of more than one possible treatment option. Actually, there are no evidence-based indications to identify the better treatment option (Athale et al. 2014). In this context, great uncertainty exists when choosing the right treatment option and SDM plays an essential role. For this reason, it is fundamental that healthcare providers encourage patients to develop an understanding of their condition and to participate actively in the decision-making, reporting their bleeding symptoms and physical status.

### Data collection and sample size

We collected data from haemophilia patients resident in Spain. The patients had the two types of haemophilia: haemophilia A or B (in haemophilia A patients do not have enough factor VIII, in haemophilia B they do not have enough factor IX). Participants in the study had to be 18 years or older. In the case of minors, we collected the information from the parent who had the most contact with the healthcare professionals.

In general, subjects were recruited through the Spanish Federation of Haemophilia and regional haemophilia organisations, which mainly by e-mail, telephone and webpage encouraged their members to participate in the study. We obtained some other participants through the Spanish Society of Haemostasis and Thrombosis, which informed haematologists through its webpage. The first participants were recruited in the XLIII National Assembly and XXI Medical-Social Symposium of the Spanish Federation of Haemophilia. The data collection lasted 4 months (from April to August 2014). Employing a convenience sampling approach we obtained a total of 181 participants, of whom 53 % are patients and the rest parents answering for their underage children affected by haemophilia. Haemophilia affects only males, so the patient sample consists of males with a mean age of 27 (SD = ±17.9). The majority of the patients (61 %) receive their pharmacological treatment very frequently (every week), 13 % less frequently (once, twice or three times a month) and 26 % infrequently (every five or more weeks).

We collected the data using an online survey. The invitation contained a link, and people agreeing to participate in the survey clicked on the link and were redirected to a webpage with the questionnaire. This helped guarantee respondent anonymity and meant we could reach a more geographically diverse population.

### Survey development

The design of the questionnaire used in the online survey was initially based on previous literature on PPSDM. Already-existing measures were used: Chen et al. (2011) for measuring healthcare professionals’ provision of information; Lee and Lin (2009) and Warren-Findlow et al. (2012) for self-efficacy; Fassaert et al. (2007) and Chen et al. (2011) for attentive listening; Briggs et al. (2011) and Camacho (2011) for proactive behaviour; and Kriston et al. (2010), Camacho (2011) and Chen et al. (2011) for PPSDM.

A preliminary version of the survey was pretested. To assure the content validity of the measures all items were discussed both with health care professionals specialized in haemophilia treatment and with researchers on the healthcare field. They were asked for their opinion on each item, to determine whether to retain it, to remove it or to make changes in the wording.

Finally, to assess the content validity of the measures, we pre-tested the resultant survey with a pilot sample of patients with haemophilia. This pre-test suggested minor changes in the wording of some questions.

### Measurement checks: reliability and construct validity

Following Churchill’s (1979) and Gerbing and Anderson’s (1988) methodological recommendations, we evaluated the psychometric properties (reliability, convergent validity and discriminant validity) of the measurement scales used. We ran a confirmatory factor analysis (CFA) using EQS 6.2.

The results of the CFA indicate that all indicators load significantly (p < 0.001) and substantively (standardized factor loadings 0.6 or above) on their respective theoretical constructs, supporting convergent validity. Reliability is evaluated using the composite reliability (CR) and the average variance extracted (AVE). These indicators exceed the recommended threshold values of 0.6 and 0.5 respectively (Table 2). Discriminant validity was also supported as the squared correlation between each pair of factors does not exceed the AVE of each.

**Table 2:** Measurement model: reliability and validity (N = 181)

Factor (F) variables	Standardised factor loading	t Student	Composite Reliability (CR)	Average Variance Extracted (AVE)	Correlations
Provision of information (F1: PI)			0.930	0.770	FI–F2: 0.759
PI1	0.821	10.139			FI–F3: 0.221
PI2	0.938	13.491			FI–F4: 0.230
PI3	0.862	12.438			F1–F5: 0.622
PI4	0.884	13.910			F2–F3: 0.193
Attentive listening (F2: AL)			0.938	0.833	F2–F4: 0.311
AL1	0.887	12.650			F2–F5: 0.655
AL2	0.942	16.611			F3–F4: 0.406
AL3	0.909	12.388			F3–F5: 0.312 F4–F5: 0.450
Self-efficacy (F3: SE)			0.901	0.754	
SE1	0.769	10.757			
SE2	0.942	12.643			
SE3	0.885	12.133			
Proactive behaviour (F4: PB)			0.815	0.595	
PB1	0.756	8.846			
PB2	0.820	10.871			
PB3	0.736	9.810			
PPSDM (F5)			0.934	0.740	
PPSDM1	0.890	13.056			
PPSDM2	0.844	10.993			
PPSDM3	0.856	12.219			
PPSDM4	0.861	14.635			
PPSDM5	0.849	13.663			

## Results

### Descriptive analysis and control variables

The measurement model was found to be satisfactory, so we then estimated the descriptive data and correlations between the different dimensions (factors). As is shown in Table 3, correlation analysis indicates that all the dimensions are positively and significatively correlated. Before evaluating the results of the structural equation model, we checked whether the dimensions differed in terms of the variables age and health literacy.^1^ Gender differences were not considered as only males are affected by haemophilia. As significant differences were not founded, the structural equation model was performed without including age or health literacy as control variables.

**Table 3:** Means, standard deviations and correlations for all scales (N = 181)

Dimensions	Mean (SD)		PI	AL	SE	PB	PPSDM
PI	4.05	(0.80)	1				
AL	4.06	(0.78)	0.720**	1			
SE	4.17	(0.70)	0.193**	0.167*	1		
PB	3.99	(0.77)	0.222**	0.284**	0.366**	1	
PPSDM	3.79	(0.81)	0.592**	0.618**	0.294**	0.408**	1

* p < 0.05; ** p < 0.001

### Proposed model

Regarding the results of the causal model, as Table 4 shows, the goodness-of-fit indices are satisfactory and four of the five proposed hypotheses are supported. According to these results, provision of information has a positive effect on self-efficacy (H1: γ_1_ = 0.230); attentive listening affects proactive behaviour (H2: γ_2_ = 0.318); self-efficacy also has a positive effect on proactive behaviour (H3: β_3_ = 0.338); and proactive behaviour has a positive effect on PPSDM (H5: β_5_ = 0.453). All the relationships considered were significative, except the one between self-efficacy and PPSDM (H4). The results cannot support this hypothesis.

**Table 4:** Results of structural equation model analyses (N = 181)

Causal relations	Standardised factor loadings (t Student)	Test
H1 (+): Provision of information (PI) → self-efficacy (SE)	0.230 (2.569)	S**
H2 (+): Attentive listening (AL) → proactive behaviour (PB)	0.318 (2.909)	S**
H3 (+): Self-efficacy (SE) → proactive behaviour (PB)	0.338 (3.613)	S**
H4 (+): Self-efficacy (SE) → PPSDM	0.135 (1.530)	NS
H5 (+): Proactive behaviour (PB) → PPSDM	0.453 (4.966)	S**

χ^2^ S–B (129) = 257.9029 (p = 0.00000), BBNNFI = 0.909, CFI = 0.923, GFI = 0.840, RMSEA = 0.075

S** supported at 95 % level, *NS* not supported

Nevertheless, as it is shown in Table 5, although self-efficacy does not have a direct effect on PPSDM (H4 rejected), an indirect effect is evident, resulting from the mediating role of proactive behaviour. These results show that provision of information and attentive listening influence PPSDM trough two intervening variables (also called mediators): self-efficacy and proactivity behaviour.^2^

**Table 5:** Total and indirect effects (N = 181)

	Standardised coefficients (t Student)
Total effect	
Provision of information (PI) → self-efficacy (SE)	0.230 (2.569)
Provision of information (PI) → proactive behaviour (PB)	0.078 (2.189)
Provision of information (PI) → PPSDM	0.066 (2.638)
Attentive listening (AL) → proactive behaviour (PB)	0.318 (2.909)
Attentive listening (AL) → PPSDM	0.144 (2.682)
Self-efficacy (SE) → proactive behaviour (PB)	0.338 (3.613)
Self-efficacy (SE) → PPSDM	0.288 (3.699)
Proactive behaviour (PB) → PPSDM	0.453 (4.966)
Specific indirect effect	
Self-efficacy (SE) → PPSDM via proactive behaviour	0.153 (3.079)

## Discussion

SDM is particularly important to research in health care and especially in chronic illness where the patients need knowledge and skills all along their lives. Following the literature about the patient perspective of SDM our research proposes a model in which the variables provision of information and attentive listening have an effect over PPSDM through two intervening variables (self-efficacy and proactivity). To the best of our knowledge no previous research have analyzed the relationship between provision of information and attentive listening on PPSDM. Patient participation on SDM depends on the communication style of the doctors (Chhabra et al. 2013) and also on patients’ preferences (Petriwskyj et al. 2014; Flynn et al. 2006). For this reason, we propose that provision of information and attentive listening do not drive directly SDM but through self-efficacy and proactivity.

Previous research have found that the more proactive is the patient the higher is his/her satisfaction with the health care system (Fullerton and McCullough 2014) and the bigger is his/her adherence to preventive health care (Kahana et al. 2010). This work shows that proactivity is a key aspect of PPSDM for patients with chronic illnesses. This is because asking for information reflects the patient’s perception that the healthcare professional is listening attentively. Besides, the doctor can see this proactive behaviour as a sign that the patient wants to participate in the decision-making. Our results suggest that the fact that the patient searches for and requests information (i.e. in the form of formulating questions or manifesting disagreement) is fundamental for them to feel they are participating in the decision-making.

Our study raises the question: why, contrary to expectations, does self-efficacy not have a direct effect on PPSDM? A possible explanation might be that self-efficacy could generate in the patient an exaggerated feeling of his or her own ability. The patients may feel independent enough to take the decision themselves, so they do not perceive the decision to be shared. However, the results obtained show that self-efficacy has an indirect and significative effect on PPSDM through proactive behaviour. Thus, self-efficacy is also a crucial factor when trying to elicit in the patient the feeling that he/she is participating in the decision-making.

These results are coherent with the studies that propose to develop communication training programs dealing with all relevant functions of communication between physicians and patients, such as SDM (de Haes and Bensing 2009; Levinson et al. 2010; Bensing et al. 2013). Thus, our work contributes to extend the idea that is essential for the doctor to establish a collaborative dialogue to encourage patient self-efficacy and proactivity in requesting information. Provision of information and attentive listening can help the patients to value their personal knowledge and expertise in relation to their doctors. These strategies are very necessary as it is widely known that “patients also place much emphasis on the medical information provided, but do not recognize, or undervalue, the complementary expertise that they can bring to the SDM encounter” (Joseph-Williams et al. 2014, p. 307).

The exchange of information in the patient–physician communication is related to characteristics of patients, characteristics of doctors and to the clinical situation (Waitzkin 1984). The same can be said with regards to SDM. Thus, it would be interesting to compare the model proposed in this work with other types of patients and to consider control variables that could be related with SDM. Among those potential control variables it should be taken into account personal characteristics (such as gender, health literacy, severity of the illness and self-advocacy) or institutional characteristics (i.e. type of institution or degree of use of communication tools such as social media or mobile apps). Moreover, it could be particularly interesting analyze the importance of empowering experience as a control variable (Suárez et al. 2016).

This research has some limitations that could be addressed in future studies. First, the cross-sectional design, that is, the data came from a specific point of time, what can affect the causality. Thus, this research could be used as a reference in future studies dealing with longitudinal data. Second, as haemophilia affects only males, the results suffer from gender bias. Future research should take into account whether there could be differences between genders in the case of other chronic illness. Finally, using an online survey is inherently problematical, in particular it is difficult to locate a representative sample (Baker et al. 2010).

## Conclusions

SDM is regarded as one of the “younger” functions in medical communication research and “patient-based studies are still in their infancy” (Bensing et al. 2013, p. 288). Recent studies call for an analysis of the antecedents of patient perceptions of SDM on PPSDM (Shay and Lafata 2015; Joseph-Williams et al. 2014). The results presented here help to bridge this gap by focusing specifically on the patient’s perspective of SDM. The proposed hypotheses are tested through SEM which is quite novel and shows high potential in the SDM domain. It should be taken into account that in this field of research it is very interesting to examine the relationship between different variables that represent patient’ self-perceptions. With this sort of variables, which should be measured as latent variables, that is, through multiple indicators, is particularly useful to apply SEM.

Our study supports previous research related with the necessity of taking into account provision of information and attentive listening as core competencies in medical education (Hartman et al. 2013; Mazzi et al. 2013). Departing from this previous studies, our research shows that provision of information and attentive listening increase PPSDM via their respective positive effects on patient self-efficacy and patient proactive behaviour in requesting information.

We would strongly recommend incorporating communication training into medical education with the aim of raising PPSDM. These training programs should focus on encouraging healthcare professionals’ attentive listening and provision of information and, at the same time, they should also consider patient self-efficacy and proactivity in requesting information.

Thus healthcare professionals can improve PPSDM by providing correct information that is personalized according to the patient’s individual circumstances. Other important skills for healthcare professionals are giving clear explanations and checking the patient’s understanding. Healthcare professionals should also create an environment of attentive listening. In other words, they should encourage patients to discuss without interruption or premature closure their main concerns, making them feel comfortable and making it easy to elicit their perceptions about their illness and associated feelings. Provision of information and attentive listening may be fundamental in helping patients not to undervalue their personal knowledge and expertise in relation to the doctor so they adopt a more active position in requesting information and in their relationship with their doctor.

It should also be highlighted that, in the sample analyzed, it has not be possible to test the direct effect of self-efficacy over PPSDM. This result suggests that patients can overvalue, or undervalue, his or her own ability. Doctors and society in general need to assimilate patient’ proactive behaviour with the idea that the patient is acting as the doctor’s partner. We believe that the patient assumes this type of behaviour when they realize that their input in the interaction with their doctor is highly valuable.

Thus interventions to raise PPSDM should not be directed solely at the healthcare professional, but should also include the patient and the people close to the patient. Specifically, another line of action is to support patients and/or the people close to them so that they are capable of taking the initiative to debate with their doctor, search for and ask for information and openly express their opinion, even their disagreement. The aim is to encourage behaviour that is responsible and proactive, respectful and directed at becoming conscious of the need to participate and feel that they are participating in the decision-making. The new information and communications technologies offer quick, convenient tools (such as blogs, social networks, and mobile phone apps) to encourage this proactive behaviour and evaluate the changes occurring in the patient’s proactive behaviour and self-efficacy.

## Abbreviations

AL: attentive listening
AVE: average variance extracted
CFA: confirmatory factor analysis
CR: composite reliability
PB: proactive behaviour
PI: information provision
PPSDM: patients’ perception of shared decision-making
SD: standard deviation
SDM: shared decision-making
SE: self-efficacy
SEM: structural equation modelling
WHO: World Health Organization

## Footnotes

1 The authors would like to thank a reviewer for suggesting the possible importance of this control variable. In this work we measured health literacy by three different indices. The results were the same in the three cases.

2 Recent statistical techniques of mediation analysis can be seen in Hayes (2009). The effect of a variable X over Y may come to be through a variety of forces both direct and indirect (this effect appears when X significative influences M and M, at the same time, significative influences Y). In this work, the EQS command Effect Decomposition was used to analyze the indirect effects. Variable M is an intervening or mediator variable.
